# Activity and sports resumption after long segment fusions to the pelvis for adult spinal deformity: survey results of AO Spine members

**DOI:** 10.1007/s43390-023-00734-6

**Published:** 2023-07-18

**Authors:** Alekos A. Theologis, Daniel D. Cummins, So Kato, Stephen Lewis, Christopher Shaffrey, Lawrence Lenke, Sigurd H. Berven, AO Spine Knowledge Forum Deformity

**Affiliations:** 1 Department of Orthopaedic Surgery, University of California–San Francisco (UCSF), 500 Parnassus Ave, MUW 3rd Floor, San Francisco, CA 94143 USA; 2 Department of Orthopaedic Surgery, The University of Tokyo, Tokyo, Japan; 3 Department of Surgery and Spine Program, University of Toronto, Toronto, ON Canada; 4 Department of Orthopaedic Surgery, Duke University, Durham, NC USA; 5 Department of Orthopedic Surgery, The Spine Hospital, Columbia University Medical Center, New York, NY USA

**Keywords:** Adult spinal deformity, Pelvic fixation, Return to sports, Survey, AO Spine

## Abstract

**Purpose:**

To assess recommendations for when adult spinal deformity (ASD) patients may return to athletic activities after surgery.

**Methods:**

A web-based survey was administered to members of AO Spine. The survey consisted of surgeon demographic information and questions asking when a patient undergoing a long thoracolumbar fusion (> 5 levels) with pelvic fixation for ASD would be allowed to resume unrestricted range of motion (ROM), non-contact sports, and contact sports postoperatively. Ordinal logistic regression was used to determine predictors for time to resume each activity.

**Results:**

One hundred twenty four members’ responses were included for analysis. The majority of respondents would allow unrestricted ROM within 3 months postop (< 3 months: 81% *vs* > 3 months: 19%]. For when to return to *non-contact* sports, the most common responses were “2–3 months” (26.6%), “3–4 months” (26.6%), and “6–12 months” (18.5%). For when to return to *contact* sports, the majority advised > 4 months postop [> 4 months: “4–6 months” (19.2%), “6–12 months” (28.0%), “ > 12 months” (28.8%) *versus* < 4 months: “1–2 months” (4.0%), “2–3 months” (1.6%), “3–4 months” (8.8%)]. 8.8% responded they would “never” allow resumption of contact sports.

**Conclusion:**

There was significant variation between surgeons’ recommendations for resumption of unrestricted range of motion and sports following long fusion with pelvic fixation for ASD. An evidence-based approach to activity recommendations will require information on outcomes and complications.

## Introduction

Surgical correction of adult spinal deformity (ASD) can offer significant improvement in quality of life (QOL) to patients [1–3]. Physical activity after ASD surgery is an important consideration with numerous implications, from short- to long-term effects on a patient’s physical health and wellbeing. In the acute postoperative period, active movement could help prevent perioperative complications such as deep vein thrombosis and pulmonary embolism [4, 5]. In the long term, maintaining and improving physical activity plays an important role in both physical and mental health [6–8].

As may be expected from spinal fixation, previous work has shown limitations in range of motion (ROM) following long spinal fusion for deformity [9, 10]. Notably for preoperative patient counseling, limited ROM may extend beyond the levels fused [10]. This limited ROM may furthermore limit strenuous physical activities and [11], for some patients, make daily activities like toileting or dressing more difficult [11, 12]. For patients who are actively engaged in sports preoperatively or who require significant ROM for their occupation, the ability to maintain ROM and activity can be particularly consequential. While some of the limitations in ROM and activities after spinal deformity surgery may be inherent to rigid fixation, encouragement of early ROM exercises and physical rehabilitation could lead to improved long-term ROM and activity in patients following surgery [11, 13]. Physician guidance can significantly influence the physical activity of patients [14]. This may be particularly true for surgeon guidance on activity postoperatively, although little work has explored patient compliance with surgeon recommendations on postoperative activity level. While evidence and guidelines exist on return to sports after surgery for adolescent idiopathic scoliosis [15], minimal evidence or guidelines exist on the recommended timeline to return to full range of motion or sports after adult spinal deformity surgery. Thus, this work’s goal is to assess recommendations on timelines for return to full range of motion and sports after adult spinal deformity surgery.

## Methods

A perioperative spine survey was formulated by a study group within AO Spine. The study group included experts in the knowledge forum (KF) degenerative and knowledge forum (KF) deformity spine. The questionnaire included demographic information on participants, including region of practice (Asian-Pacific, Europe/Southern Africa, Latin-America, Middle East/North Africa, and North America), gender (male or female), age of the surgeon (25–34, 35–44, 45–54, 55–64, or 65 +), years in practice (< 5, 5–10, 11–15, 16–20, > 20), specialty (neurosurgery or orthopedic surgery), practice setting (academic, private practice, or public), fellowship training status, and annual case volume (< 50, 51–100, 101–150, 151–200, or 201–250, or > 250). The survey was designed to cover various aspects of perioperative care such as wound management, antibiotics, bracing, and activity instructions. An online survey was distributed via email to AO Spine users and members between March 3 and March 22, 2022. The survey was targeted at surgeons performing at least ten cases per year using one or more of the following procedures:

- Long fusion (> 5 levels) for adult spine deformity patients extending to pelvis
- Long fusion (> 5 levels) for adult spine deformity patients NOT extending to pelvis
- Open 1 or 2 level fusion for adult lumbar degenerative pathologies
- MIS 1 or 2 level fusion for adult lumbar degenerative pathologies
- Open 3 to 5 level fusion for adult lumbar degenerative pathologies

It was estimated that over 6000 surgeons that were AO Spine users and members received the email. Among all those who received the email, 354 responded and 280 completed the survey. Of the surgeons who completed the survey, 164 performed adult spine deformity operations (procedures A and/or B) and 261 performed adult spinal degenerative operations (procedures C, D and/or E).

In the present study, 124 members responded to questions about when (immediately after surgery, 4–8 weeks, 8–12 weeks, 12–16 weeks, 4–6 months, 6–12 months, > 12 months, never) patients who underwent long thoracolumbar fusion (> 5 levels) with pelvic fixation for ASD would be allowed to resume unrestricted range of motion, non-contact sports, and contact sports postoperatively.

### Statistical analysis

All statistical comparisons were performed using R version 4.2.1. Ordinal logistic regression was used to determine significant predictors for recommended time after surgery to unrestricted range of motion, return to non-contact sport, and return to contact sport.

## Results

### Survey respondents

One hundred twenty four members’ responses were included for analysis (Table 1**)**. Most respondents were male (99.2%) with fellowship training (66.1%) and from orthopedic surgery (81.4%). Respondents were mixed with regard to years in practice (< 5 yrs: 14.4%; 5–10 yrs: 18.4%; 11–15 yrs: 22.4%; 16–20 yrs: 20.0%; > 20 yrs: 24.8%), location of practice (North America—16.8%, Latin America—38.4%; Europe/Southern Africa—38.4%; Middle East/Northern Africa—11.2%; Asia Pacific—20.8%), and type of practice (Academic—52.8%; Public/Military—25.6%; Private—20.8%).

**Table 1:** Survey responses for return to activity after adult spinal deformity surgery

	*N* (%)
Return to unrestricted range of motion	
Immediately after surgery	32 (25.8)
4–8 weeks	34 (27.4)
8–12 weeks	34 (27.4)
12–16 weeks	16 (12.9)
> 16 weeks	7 (5.6)
Return to non-contact sports	
Immediately after surgery	4 (3.2)
4–8 weeks	14 (11.3)
8–12 weeks	33 (26.6)
12–16 weeks	33 (26.6)
4–6 months	14 (11.3)
6–12 months	23 (18.5)
Refrain from sports	1 (0.8)
Return to contact sports	
Immediately after surgery	1 (0.8)
4–8 weeks	5 (4.0)
8–12 weeks	2 (1.6)
12–16 weeks	11 (8.8)
4–6 months	24 (19.2)
6–12 months	35 (28.0)
> 12 months	36 (28.8)
Refrain from sports	11 (8.8)
Region	
Asia Pacific	26 (20.8)
Europe/Southern Africa	48 (38.4)
Latin America	16 (12.8)
Middle East/North Africa	14 (11.2)
North America	21 (16.8)
Gender	
Male	124 (99.2)
Female	1 (0.8)
Age	
25–34	8 (6.4)
35–44	48 (38.4)
45–54	41 (32.8)
55–64	25 (20.0)
65 +	3 (2.4)
Years in practice	
< 5	18 (14.4)
5–10	23 (18.4)
11–15	28 (22.4)
16–20	25 (20.0)
> 20	31 (24.8)
Specialty	
Neurosurgery	24 (19.2)
Orthopedic surgery	101 (80.8)
Practice setting	
Academic	66 (52.8)
Private practice	32 (25.6)
Public	26 (20.8)
Fellowship	
No	84 (67.2)
Yes	41 (32.8)
Case volume	
< 50	2 (1.6)
51–100	25 (20)
101–150	27 (21.6)
151–200	28 (22.4)
201–250	14 (11.2)
> 250	29 (23.2)

**Fig. 1:**
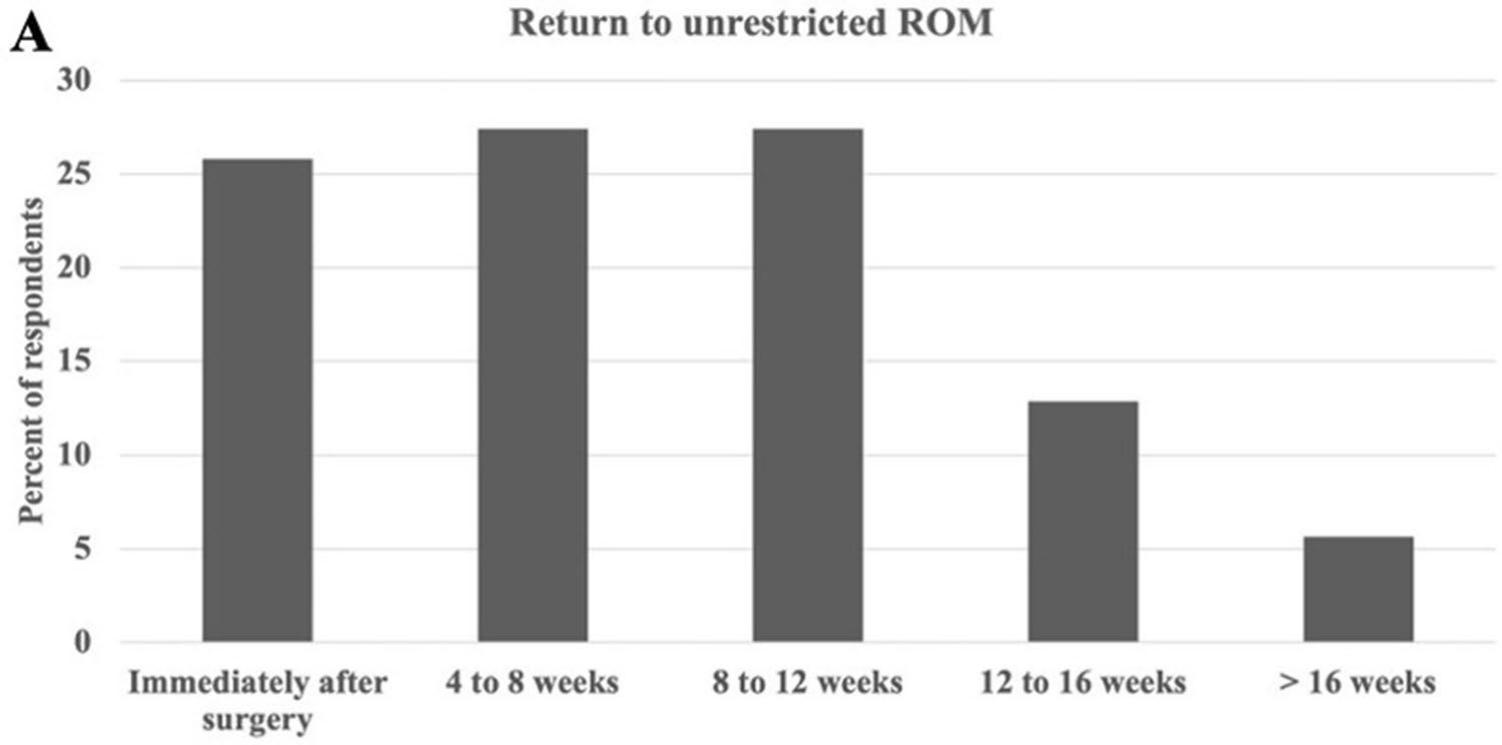
Survey results by percentage of AO Spine member respondents for return to unrestricted range of motion (ROM) following multi-level instrumented fusions to the pelvis for adult spinal deformity

The majority of respondents would allow unrestricted ROM within 3 months postop [< 3 months: “immediately after surgery” (25.8%), “1–2 months” (27.4%), and “2–3 months” (27.4%) *versus* > 3 months: “3–4 months” (12.9%) and “ > 4 months” (5.6%)] (Fig. 1). Asian-Pacific region responders recommended later return to unrestricted ROM compared to European (odds ratio (OR) = 0.28), Latin-American (OR = 0.27), and North American (OR = 0.30) respondents, *p* < 0.05. Other predictors of shorter return to unrestricted ROM were male respondents (OR = 0.03; *p* < 0.05) and fellowship training (OR = 0.36; *p* < 0.05) (Table 2).

**Table 2:** Predictors of recommendation for unrestricted range of motion

Covariate	Univariate analysis		Multivariate analysis	
	OR (95% CI)	*p *value	OR (95% CI)	*p* value
Region Asia Pacific Europe/Southern Africa Latin America Middle East/North Africa North America	Reference 0.43 (0.18–1.00) 0.45 (0.16–1.30) 0.48 (0.16–1.49) 0.39 (0.14–1.13)	Reference 0.051 0.140 0.205 0.083	Reference 0.28 (0.11–0.73) 0.27 (0.08–0.90) 0.47 (0.13–1.68) 0.30 (0.09–0.98)	Reference 0.0089 0.033 0.243 0.047
Gender	N/A	N/A	N/A	N/A
Age 25–34 35–44 45–54 55–64 65 +	Reference 1.13 (0.31–4.12) 0.80 (0.22–2.99) 0.54 (0.13–2.21) 1.28 (0.11–14.29)	Reference 0.851 0.745 0.390 0.842	Reference 3.78 (0.54–2.65) 3.96 (0.42–3.73) 3.70 (0.32–42.15) 1.23 (0.45–33.31)	Reference 0.181 0.228 0.292 0.136
Years in Practice < 5 5–10 11–15 16–20 > 20	Reference 0.45 (0.15–1.33) 0.60 (0.20–1.78) 0.67 (0.22–2.04) 0.64 (0.20–2.03)	Reference 0.450 0.483 0.355 0.150	Reference 0.42 (0.08–2.40) 0.43 (0.07–2.52) 0.32 (0.04–2.23) 0.22 (0.03–1.65)	Reference 0.334 0.248 0.341 0.142
Specialty Neurosurgery Orthopedic surgery	Reference 0.64 (0.29–1.40)	Reference 0.263	Reference 0.69 (0.27–1.75)	Reference 0.428
Practice setting Academic Private practice Public	Reference 1.72 (0.76–3.88) 1.06 (0.50–2.25)	Reference 0.194 0.887	Reference 2.06 (0.80–5.28) 1.02 (0.44–2.32)	Reference 0.135 0.971
Fellowship No Yes	Reference 0.40 (0.21–0.79)	Reference 0.0083	Reference 0.36 (0.16–0.81)	Reference 0.013
Case volume < 50 51–100 101–150 151–200 201–250 > 250	Reference 1.67 (0.11–25.67) 2.81 (0.18–43.15) 2.17 (0.14–33.19) 1.19 0.07–20.16) 2.32 (0.15–35.48)	Reference 0.712 0.906 0.578 0.458 0.546	Reference 2.30 (0.13–4.05) 2.64 (0.15–47.14) 2.73 (0.16–47.00) 1.10 (0.05–23.61) 2.50 (0.14–45.13)	Reference 0.568 0.509 0.490 0.951 0.534

*CI* confidence interval, *OR* odds ratio, *N/A* not available

**Fig. 2:**
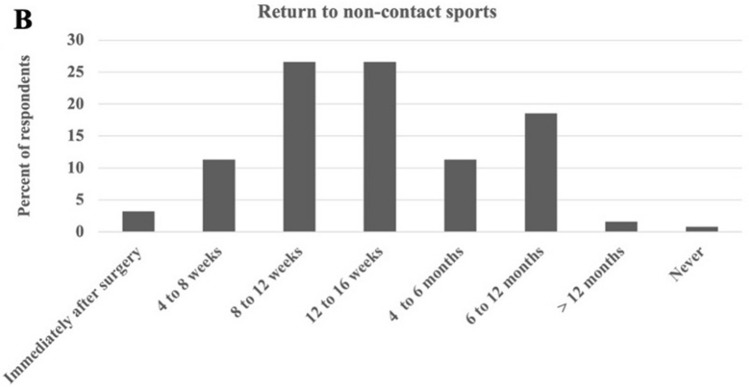
Survey results by percentage of AO Spine member respondents for return to non-contact sports following multi-level instrumented fusions to the pelvis for adult spinal deformity

For when to return to *non-contact* sports, the most common responses were “2–3 months” (26.6%), “3–4 months” (26.6%), and “6–12 months” (18.5%). Infrequent responses were “1–2 months” (11.3%), “4–6 months” (11.3%), “immediately after surgery” (3.2%), " > 12 months” (1.6%), and “never” (0.8%) (Fig. 2**)**. Asian-Pacific responders recommended later return to unrestricted ROM compared to European (OR = 0.33), Latin-American (OR = 0.12), and North American (OR = 0.27) respondents (*p* < 0.05). Older age of the surgeon was a predictor of later return to non-contact sports (OR = 10.93; *p* < 0.05), Table 3.

**Table 3:** Predictors of return to non-contact sport

Covariate	Univariate analysis		Multivariate analysis	
	OR (95% CI)	*p* value	OR (95% CI)	*p* value
Region Asia Pacific Europe/Southern Africa Latin America Middle East/North Africa North America	Reference 0.35 (0.15 – 0.82) 0.12 (0.04 – 0.38) 0.33 (0.10 – 1.07) 0.27 (0.10 – 0.78)	Reference 0.015 2.39E-4 0.064 0.015	Reference 0.33 (0.13 – 0.81) 0.12 (0.03 – 0.44 0.35 (0.09 – 1.29) 0.27 (0.08 – 0.92)	Reference 0.016 0.0014 0.113 0.036
Gender	N/A	N/A	N/A	N/A
Age 25–34 35–44 45–54 55–64 65 +	Reference 6.12 (1.57 – 24.14) 4.14 (1.06 – 16.25) 6.93 (1.60 – 29.94) 19.63 (0.92 – 417.83)	Reference 0.009 0.042 0.010 0.056	Reference 7.28 (1.14 – 46.61) 4.90 (0.58 – 41.29) 10.93 (1.05 – 114.12) 20.64 (0.48 – 887.54)	Reference 0.036 0.144 0.046 0.115
Years in practice < 5 5–10 11–15 16–20 > 20	Reference 1.78 (0.55 – 5.74) 1.73 (0.58 – 5.12) 1.33 (0.45 – 3.94) 2.55 (0.86 – 7.54)	Reference 0.337 0.325 0.609 0.091	Reference 0.76 (0.46 – 2.80) 0.96 (0.22 – 4.19) 0.77 (0.15 – 4.04) 0.98 (0.17 – 5.61)	Reference 0.718 0.955 0.755 0.979
Specialty Neurosurgery Orthopedic surgery	Reference 1.39 (0.64 – 3.01)	Reference 0.401	Reference 1.13 (0.46 – 2.80)	Reference 0.785
Practice setting Academic Private practice Public	Reference 1.03 (0.45 – 2.36) 1.05 (0.50 – 2.18)	Reference 0.951 0.903	Reference 1.35 (0.51 – 3.54) 0.89 (0.40 – 2.01)	Reference 0.545 0.779
Fellowship No Yes	Reference 1.14 (0.59 – 2.20)	Reference 0.706	Reference 1.03 (0.48 – 2.22)	Reference 0.945
Case volume < 50 51–100 101–150 151–200 201–250	Reference 2.38 (0.26 – 22.06) 5.08 (0.56 – 46.25) 2.09 (0.23 – 18.80) 2.02 (0.20 – 20.65)	Reference 0.446 0.150 0.509 0.555	Reference 5.70 (0.45 – 130.81) 10.41 (0.83 – 42.48) 5.32 (0.45 – 62.27) 2.92 (0.20 – 42.48)	Reference 0.179 0.070 0.183 0.432

*CI* confidence interval, *OR* odds ratio, *N/A* not available

**Fig. 3:**
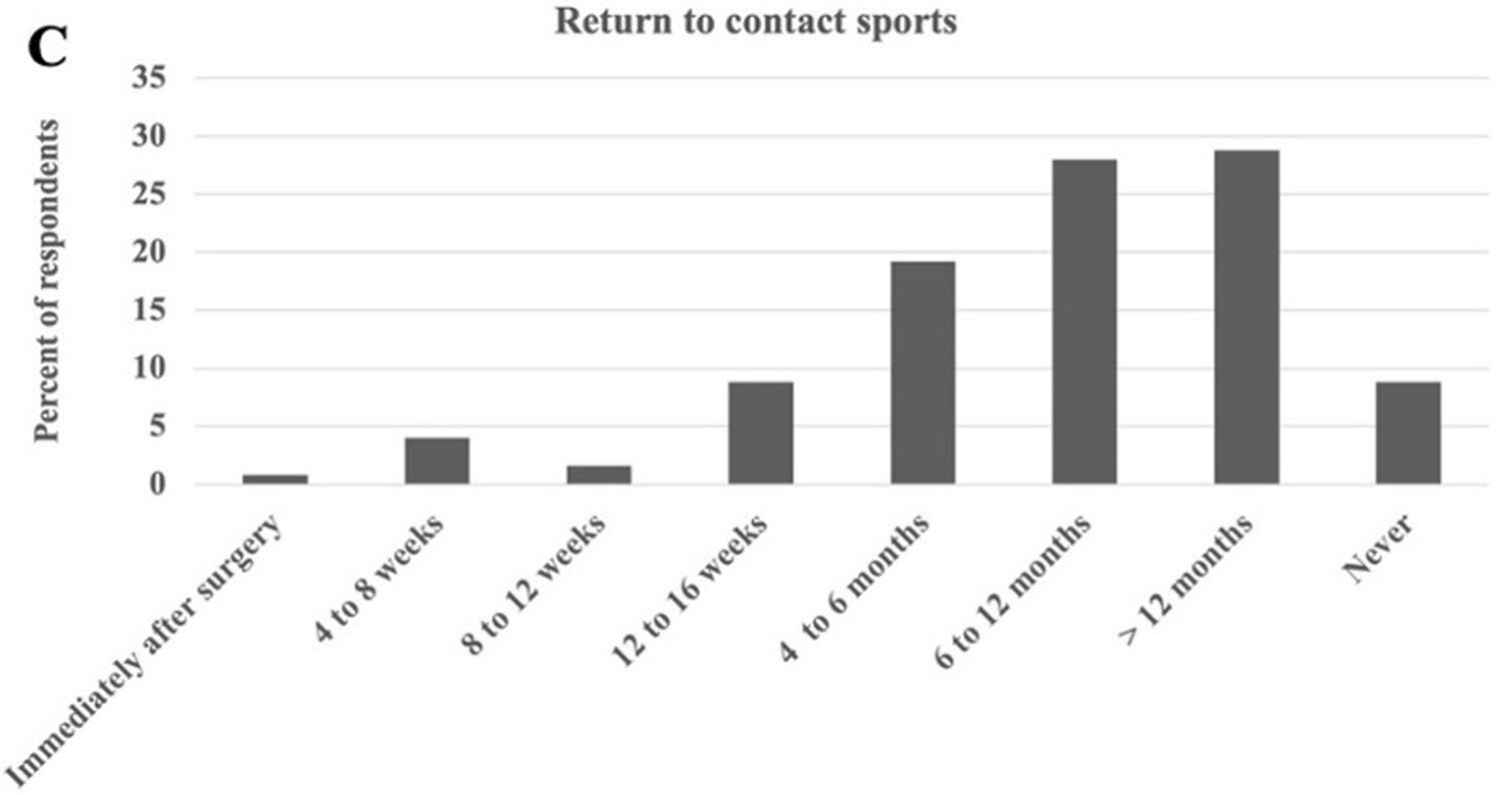
Survey results by percentage of AO Spine member respondents for return to contact sports following multi-level instrumented fusions to the pelvis for adult spinal deformity

For when to return to *contact* sports, the majority advised > 4 months postop [> 4 months: “4–6 months” (19.2%), “6–12 months” (28.0%), “ > 12 months” (28.8%) *versus* < 4 months: “1–2 months” (4.0%), “2–3 months” (1.6%), “3–4 months” (8.8%)]. 8.8% responded they would “never” allow resumption of contact sports (Fig. 3). Respondents from Asian-Pacific regions recommended later return to unrestricted ROM compared to European (odds ratio (OR) = 0.13), Latin-American (OR = 0.04), and North American (OR = 0.08) respondents (*p* < 0.05) (Table 4**)**.

**Table 4:** Predictors of return to contact sport

Covariate	Univariate analysis		Multivariate analysis	
	OR (95% CI)	*p* value	OR (95% CI)	*p* value
Region Asia Pacific Europe/Southern Africa Latin America Middle East/North Africa North America	Reference 0.20 (0.04 – 0.95) 0.04 (0.01 – 0.22) 0.24 (0.04 – 1.48) 0.09 (0.02 – 0.46)	Reference 0.043 2.27E-4 0.123 0.004	Reference 0.13 (0.02 – 0.70) 0.04 (0.06 – 0.25) 0.22 (0.25 – 1.89) 0.08 (0.01 – 0.49)	Reference 0.018 6.50E-4 0.167 0.006
Gender	N/A	N/A	N/A	N/A
Age 25–34 35–44 45–54 55–64 65 +	Reference 0.68 (0.12 – 3.75) 0.60 (0.11 – 3.36) 0.95 (0.15 – 5.88) 0.44 (0.02 – 9.18)	Reference 0.655 0.563 0.953 0.595	Reference 1.94 (0.09 – 42.95) 2.62 (0.09 – 78.51) 4.31 (0.12 – 155.25) 0.59 (0.06 – 59.89)	Reference 0.675 0.579 0.425 0.824
Years in practice < 5 5–10 11–20 16–20 > 20	Reference 0.34 (0.08 – 1.51) 0.42 (0.10 – 1.81) 0.32 (0.07 – 1.39) 0.75 (0.17 – 3.34)	Reference 0.157 0.245 0.129 0.709	Reference 0.14 (0.01 – 1.85) 0.12 (0.01 – 1.76) 0.15 (0.01 – 2.58) 0.30 (0.02 – 5.37)	Reference 0.135 0.122 0.194 0.414
Specialty Neurosurgery Orthopedic surgery	Reference 1.68 (0.71 – 3.95)	Reference 0.239	Reference 1.36 (0.43 – 4.27)	Reference 0.598
Practice setting Academic Private practice Public	Reference 0.46 (0.19 – 1.14) 1.13 (0.45 – 2.83)	Reference 0.095 0.787	Reference 0.49 (1.54 – 1.53) 0.68 (0.23 – 2.05)	Reference 0.219 0.494
Fellowship No Yes	Reference 0.75 (0.35 – 1.63)	Reference 0.473	Reference 0.69 (0.24 – 2.00)	Reference 0.500
Case volume < 50 51–100 101–150 151–200 201–250 > 250	Reference 1.78 (0.20 – 46.06) 7.98 (0.48 – 132.33) 1.56 (0.11 – 22.91) 1.71 (0.11 – 27.76) 3.03 (0.20 – 46.06)	Reference 0.676 0.147 0.748 0.706 0.425	Reference 6.35 (0.32 – 1.27) 33.87 (1.35 – 847.91) 3.37 (0.18 – 6.28) 5.84 (0.26 – 130.02) 5.84 (0.30 – 111.95)	Reference 0.227 0.032 0.415 0.265 0.242

*CI* confidence interval, *OR* odds ratio, *N/A* not available

## Discussion

Physical activity is a crucial element of an individual’s quality of life and physical health. As such, return to physical activity should be a primary concern in the postoperative care of patients who undergo spinal fusion for adult spinal deformity. Despite this, there remains no established guidelines on time to return to unrestricted range of motion, non-contact sports, and contact sports postoperatively in this patient population. Furthermore, the presented results of recommendations from AO Spine members suggest significant variation in practice patterns among spine surgeons. Some overarching trends did emerge in recommended time to return to activity: the majority (81.5%) of survey respondents recommend return to unrestricted range of motion within 3 months and the majority (84.8%) recommended at least 4 months until return to contact sports. There was much greater variation in return to non-contact sports.

### Unrestricted range of motion after ASD surgery

The majority of AO Spine members (81.5%) recommended return to unrestricted range of motion within 3 months of ASD surgery. The primary tradeoffs in time to unrestricted ROM are the benefits of early movement versus the risk of hardware complications from premature movement before full fusion. Although early movement after spinal surgery as part of Enhanced Recovery after Surgery (ERAS) protocols may lead to shorter length of stay, reduced postoperative pain, and decreased complication rates [16], little work has demonstrated the particular role of early movement after spine surgery, particularly for adult spinal deformity [17]. Conversely, excessive ROM proximal to long spinal constructs has been shown to increase rates of mechanical failure [18], which can lead to significant complications necessitating revision operations. However, pseudarthrosis may occur well after 1 year following spinal fusion [19]. Thus, the trend to recommend resuming unrestricted range of motion after 3 months from ASD surgery lacks strong evidence.

### Non-contact sports after ASD surgery

There is a paucity of data on return to non-contact sports after spinal deformity surgery in adults. In adolescents, the majority of surgeons permit return to non-contact sports by 3 months postoperatively [20, 21]. In a prospective study on 26 athletes treated with posterior spinal fixation for adolescent idiopathic scoliosis, return to sports occurred at a median time of 2.7 months without complication. The main barriers to return to sports were physical conditioning and flexibility, rather than surgeon guidance against activity [22]. This approximates the majority of AO Spine respondents who recommended return to non-contact sports within 4 months for adult patients. However, there were still a substantial minority who delayed activity beyond this time, which may delay beneficial effects of sports on physical and mental health for patients.

### Contact sports after ASD surgery

The recommendations for time to return to contact sports were expectedly more conservative compared to other activities. The vast majority of AO Spine members (84.8%) recommended waiting at least 4 months after surgery to return to contact sports. Previous literature has suggested radiographic assessment of construct fusion prior to returning to contact sports [20, 23], which underscores an individualized approach to return to contact sports. The precise contact sport may also be relevant. For instance, approximately 20% of surgeons recommend permanently refraining from “collision” sports, including American football, hockey, rugby, and mixed martial arts, as opposed to other contact sports such as soccer, basketball, or volleyball [21]. One limitation of the present study is not defining exact activities in each category or distinguishing collision sports from other contact sports. Notably, fewer AO Spine members (8.8%) recommended permanently avoiding contact sports after adult spinal deformity surgery with pelvic fixation.

### Variables associated with return to activity after surgery

Asian-Pacific AO Spine members had significantly more conservative recommended times to resume unrestricted range of motion compared to European/Southern African (OR = 0.28; 95% CI = 0.11–0.73; *p* = 0.0089), Latin-American (OR = 0.27; 95% CI = 0.08–0.90; *p* = 0.033), or North American (OR = 0.30; 95% CI = 0.09–0.98; *p* = 0.047) members. Asian-Pacific members were also more conservative in time to return to both non-contact and contact sports. While the exact reasons for this difference are unclear, it may be secondary to prevailing osteoporosis in Asian-Pacific patients and surgeons' concern about it. These variations indicate regional practice patterns prevailing over evidence-based practice. Fellowship-trained members also recommended shorter time to return to unrestricted ROM (OR = 0.36; 95% CI = 0.16–0.91; *p* = 0.013) despite a lack of evidence-based guidelines. Other results including member/surgeon age and case volume were less clear.

The results of this study should be considered in the context of its limitations. The most notable limitation is the low response rate of the survey (~ 5%), which introduces the very real possibility of considerable selection bias among the respondents. As such, the respondents’ responses may not be representative of the larger spine community as a whole. This is magnified by the fact that the data are purely opinion based and are probably highly influenced by the training of the respondent surgeons and the preferences of their mentors and teachers, as there are no clear data about return to activity levels and the appropriateness in adult deformity patients. While the response rate is low, it is in concordance with multiple prior published survey studies from the AO related to spinal pathologies [24–27]. What also cannot be gleamed from this survey are the specific reasons for the respondents’ responses. Knowing whether the surgeons are protecting themselves from a critique of their own failure or are they protecting the patients from the patient themselves or simply choosing to limit the patients would strengthen our ultimate understanding of return to activity and sport recommendations following surgery. Along the same vein, it raises the philosophical question of whether the patients have the right to determine their own activity and risk level or not following surgery? Another key limitation is that the survey did not provide more granular information on patient factors (i.e., age, DEXA scores, fitness levels, frailty, psychiatric profiles) and specific surgical details (i.e., exact levels of fusion, instrumentation/rod materials, interbody support, cement augmentation, alignment), both of which likely play roles in determining when a surgeon may feel a patient is suitable to return to certain types of activities. Given the heterogeneity of patients who undergo operative intervention for adult spinal deformity as well as the associated surgical strategies, future studies on this topic would benefit from prospective designs that query more specific real-world case scenarios so as to further differentiate between timelines for return to sport after surgery for ASD. While a more rigorous study design in which establishing criterion and then following a prospective cohort would provide more clinically relevant data, the current study will hopefully serve as a benchmark from which additional studies will be designed focusing on defining appropriate criteria and recommendations on timing of return to activities and sports for patients following long segment fusions for adult spinal deformity.

## Conclusion

Physical activity is an essential component of physical and mental health, and is an important consideration after surgery for adult spinal deformity. There remains a lack of data and evidence-based guidelines on return to activities after surgery for adult spinal deformity. There is a need for prospective research regarding the impact of postoperative activity limitation on changing mechanical complications and health status after adult spinal deformity surgery.

## Data Availability

Data available on request from the authors.
